# A self-disclosure model for adolescents with perinatally acquired HIV in Eswatini

**DOI:** 10.4102/curationis.v48i1.2741

**Published:** 2025-07-31

**Authors:** Baliwe P. Dlamini, Ntombifikile G. Mtshali

**Affiliations:** 1 School of Nursing and Public Health, College of Health Sciences, University of KwaZulu-Natal, Durban, South Africa

**Keywords:** adolescents, disclosure efficacy, Eswatini, HIV, model, self-disclosure

## Abstract

**Background:**

Adolescents living with HIV (ALHIV) are faced with the hard decision of how to disclose their HIV status to others. Despite this obvious challenge, few HIV self-disclosure models exist.

**Objectives:**

This study was aimed at developing a self-disclosure model that would assist adolescents with perinatally acquired HIV in Eswatini to share their HIV status with others.

**Method:**

The explanatory sequential mixed method design was used; therefore, quantitative data were collected first from 361 ALHIV aged 15–19 years using questionnaires and analysed. Qualitative data were collected from 23 ALHIV, 24 nurses and 4 policymakers using 3 focus group discussions and in-depth individual interviews. For quantitative data, statistical analysis was utilised, and grounded theory guided the analysis of qualitative data and the development of the model.

**Results:**

HIV self-disclosure is the central concept for this model, and it is supported by four major concepts: (1) national HIV strategic framework, (2) enablers, (3) adolescent empowerment and (4) model outcomes. The description of the model was conducted using Chinn and Kramer’s stages of model development.

**Conclusion:**

HIV education and social support are important in reducing stigma and discrimination in communities where adolescents reside. Prioritising the training of nurses and developing self-disclosure guidelines would lead to a remarkably increased level of adolescent HIV self-disclosure.

**Contribution:**

The contribution of this study is that it is the first of its kind to develop an adolescent HIV self-disclosure model in Eswatini.

## Introduction

HIV self-disclosure remains a challenge affecting adolescents living with HIV (ALHIV) (Khan, Garman & Sorsdahl 2023). Adolescents are not eager to disclose their status to others (Hogwood, Campbell & Butler 2013; Mburu et al. 2014) and consider the disclosure process to be complex, much like adults (Siu et al. 2012). Nonetheless, they ultimately disclose to friends, romantic partners or teachers when they feel it is unavoidable (Madiba & Mokgatle 2016). HIV status disclosure is vital for varied reasons, and it is beneficial for ALHIV because it promotes mental well-being, enables them to receive social support, promotes adherence to treatment and decreases unprotected sexual intercourse (Evangeli & Foster 2014; Thoth et al. 2014).

Consequently, even though disclosure diminishes the psychological effects of managing a chronic illness discretely (Olumide & Owoaje 2020) and is important for access to social support, the fear of discrimination, stigma and rejection by peers and romantic partners prevents ALHIV from disclosing their status (Hogwood et al. 2013; Mburu et al. 2014; Midtbø et al. 2012). Other factors preventing adolescents from disclosing their status to others include a lack of communication skills and self-efficacy (Gabbidon et al. 2020). Adolescents regard information about their HIV status as private (Madiba & Mokgatle 2016; Siu et al. 2012) and wish for autonomy to reveal their status to others on their terms (Madiba & Mokgatle 2016). Previous studies indicate that within sexual relationships adolescents have a higher possibility to disclose when there is trust that is determined by the duration and connection within the relationship (Hogwood et al. 2013) and when adolescents are older, have known about their HIV status for longer and when they have had positive disclosure experiences themselves (Fair & Albright 2012).

Eswatini had 10 153 ALHIV in 2021 who received care through diverse anti-retroviral therapy service delivery models (Hlophe et al. 2025b). In Eswatini, the stigma surrounding HIV and AIDS keeps many people from getting tested for the virus or disclosing their status. People living with HIV are frequently ostracised because HIV is thought to be associated with sexual promiscuity (UNICEF 2018). Swati ALHIV also experience stigma and discrimination from their families, peers and friends (Hlophe, Nyasulu & Shumba 2025a; Nsibandze et al. 2020). Anti-retroviral therapy adherence is significantly low among Swati ALHIV, which could promote onward transmission of HIV to sexual partners (Hlophe et al. 2025a). Swati ALHIV have a low rate of HIV status self-disclosure (22%), and according to adolescents, they felt that, like any other illness, their HIV status was a personal matter and that they did not need to tell their peers or sexual partners about it (Dlamini & Mtshali 2023). In 2006, the Baylor Center of Excellence collaborated with the Ministry of Health (MoH) to facilitate teen clubs in Eswatini (Dlamini 2023) (unpublished). The age of enrolment to the teen club is 10 years, and it is a requirement that every child is disclosed to before they are enrolled at the teen club. Notably, the program was successful in promoting treatment adherence among ALHIV and primary disclosure, which is crucial in accepting one’s HIV status (UNICEF 2018).

According to literature, several HIV self-disclosure models and theories exist; however, these self-disclosure models used adult participants and were not specifically tailored for ALHIV, which is considered a unique population; the Disclosure Processes Model (DPM) posits that it is important to understand ‘*when*’ and ‘*why*’ People Living with HIV (PLHIV) feel the need to self-disclose (Chaudoir & Fisher 2010). In the DPM, a disclosure event is defined as the verbal communication that happens between the discloser and a confidant concerning the previously concealed stigmatised condition, and this disclosure event culminates with both individuals having reached an agreement about understanding the shared information (Chaudoir & Fisher 2010). A review and application of the initial DPM posited that antecedent goals affect the possibility of disclosure in a given situation, as disclosure is regarded as a goal-directed behaviour whereby individuals may disclose because they wish to improve intimacy in their relationships (Chaudoir, Fisher & Simoni 2011).

The disease progression theory (DPT), which aimed to analyse the applicability of two HIV disclosure theories in the United States, postulates that individuals disclose their HIV status when HIV progresses and they start to exhibit clinical symptoms (Serovich, Lim & Mason 2008). On the contrary, non-disclosure may result from an individual wishing to lead a normal life and prevent distress to those close to that individual (Serovich et al. 2008).

The DPM considers disclosure as a once-off event, whereas drawing from previous literature (Damian et al. 2019; Khan et al. 2023), disclosure is regarded as an ongoing process rather than a single event. Despite accounts providing an explanation of the goals that may impact disclosure decisions, the DPM does not clarify what preparation the PLHIV requires ahead of the disclosure event. The DPT posits that individuals disclose when they become symptomatic and does not consider that they may disclose because of other reasons besides disease progression. According to literature, individuals living with HIV may disclose for various reasons, and these could be for enhanced well-being, decreased feelings of isolation, increased social support and a sense of loyalty to the chosen confidant (Galano et al. 2017; Thoth et al. 2014). This theory also does not focus on the ‘process’ of disclosure from the moment the individual discloses the status up to how the confidant receives and processes the information about the status. The documented self-disclosure models are resource intensive because of the nature of being longitudinal and thus unsuitable for a resource-constrained country like Eswatini and do not recognise other contextual influences that can impact the disclosure process. Therefore, this study aimed to develop a self-disclosure model that is contextually relevant and would assist ALHIV in Eswatini to disclose their status to others.

## Research methods and design

### Research design

The study used the explanatory sequential mixed method design, and model development was guided by grounded theory. Based on the chosen mixed method design, quantitative data were collected first from ALHIV and analysed. Findings that emerged from the quantitative study assisted in highlighting areas that needed further exploration during qualitative data collection (Creswell & Creswell 2018) and helped in the formulation of the types of qualitative questions to ask participants in the qualitative phase. The overall intent of this design is to have the qualitative data help to provide more depth and insight into the quantitative results (Creswell & Creswell 2018). In this study, integration, which is defined as the clear interconnection of the quantitative and qualitative elements of a mixed methods study and is a vital and defining feature of mixed methods research, occurred at various stages: study design level that entails collecting and analysing quantitative data, after which the findings inform subsequent qualitative data collection and analysis, at methods level through connecting when one form of data connects with the other through a sample frame and interpretation and reporting level where data were integrated using joint presentations after it was brought together visually to acquire new insights beyond what is received from the distinct quantitative and qualitative results (Fetters, Curry & Creswell 2013).

Two questions from the mixed method alphabet by ErtesvAag, Sammons & Blossing (2021) were used to inform the data integration process, and these were: Q. What attempts were made to link or integrate the qualitative and quantitative components of the study? To integrate the qualitative and quantitative findings, data were visually presented in a table to highlight qualitative findings that corroborate with quantitative findings. The second question from the mixed method alphabet was: Y. Do the researchers make new knowledge claims that are based on the integration and synthesis of qualitative and quantitative data and findings? In this study, this was achieved through joint display, where data integration was performed by bringing the data together to gain new insights beyond the information gained from the separate quantitative and qualitative results and come up with new concepts (Fetters et al. 2013) as depicted in a table. As the study culminated in the development of a model, the questions also assisted in identifying the types of links arising to influence the structure of the theory and the emerging concepts and then informed model development (Chinn & Kramer 2011).

### Research setting

Data were collected from 10 sites. The sites are situated in four Eswatini regions. Regional hospitals chosen were two, health centres were four and clinics were four. All chosen sites provide paediatric and adult HIV care and treatment services. Cluster sampling was used to sample facilities for the study, and because of the uneven distribution of facilities across the four regions, more facilities came from Hhohho (3 sites) and Manzini (3 sites) regions, while the least number came from Lubombo (2 sites) and Shiselweni (2 sites) regions.

### Study population and sampling strategy

The population in this study were adolescents with perinatally acquired HIV between the ages of 15–19 who already knew their status and were taking Anti-Retroviral Therapy (ART) State Registered Nurses working in the ART clinics and policymakers involved in the enactment of policies that address HIV prevention and treatment needs of adolescents in Eswatini. **Anti-Retroviral Therapy** is medical treatment containing a variety of anti-retroviral drugs to suppress HIV and stop HIV progression (World Health Organization, 2016). Adolescents living with HIV, nurses and policymakers made up the study population. In the quantitative phase, 361 adolescents between the ages of 15 and 19 who were aware of their status were enlisted. Enrolment in HIV care at the age of 10 years was used to classify adolescents as perinatally infected. The inclusion criteria for the study were: being a male or female adolescent aged 15–19 years, adolescents who are aware of their HIV status for a period of at least 6 months, adolescents perinatally infected with HIV, taking ART for at least 6 months, State Registered Nurses working in ART clinics for 2 years who have been trained in basic Integrated Management of Adolescent and Adult Illness (IMAI) and Nurse Led Antiretroviral Therapy Initiation in Eswatini (NARTIS) and policymakers who have served in their current position for 1 year and above. The exclusion criteria for the study were: ALHIV who have not been disclosed to, adolescents not living with HIV, adolescents aged below 15 years, adolescents who are not Teen Club members, ALHIV < 18 without caregivers and nurses not involved in direct care of ALHIV. From among the eligible population, participants were chosen by simple random sampling. Each potential participant was given a unique number, and numbers were drawn starting at one until the sample size reached 361, as shown in Table 1. About 10% was added to the sample size and rounded up to 400, to account for refusals, non-responses and incomplete questionnaires. The response rate was 90%, and a total of 13 questionnaires were discarded because they were incomplete.

**TABLE 1:** Sample size for adolescents in the quantitative phase.

Site	Sample size
Site A	33
Site B	55
Site C	41
Site D	31
Site E	26
Site F	57
Site G	55
Site H	23
Site I	21
Site J	19

**Total**	**361**

*Source:* Adapted from Dlamini, B.P. & Mtshali, N.G., 2024b, ‘Views of adolescents living with perinatally acquired HIV on HIV status disclosure in Eswatini’, *AIDS Care* 36(9), 1263–1271. https://doi.org/10.1080/09540121.2023.2299334

The sample size was calculated using Equation 1:


$$n=\frac{Z^{2}\times (p)\times (1-p)}{e^{2}}$$ [Eqn 1]

where:

*Z* value is 1.96 for 95% confidence interval (CI)

*p* = 0.5

*e* = 0.05

For the qualitative phase, the population was made up of 23 ALHIV, 24 nurses and 4 policymakers. Purposive sampling was used to recruit participants in this study, as those participants considered to have rich data were invited to be part of the study. These were adolescents whose responses provided deeper insights into their narratives and experiences during interviews and nurses and policymakers with vast experience in their field. For adolescents, the qualitative database was linked to the quantitative database through sampling. All sites were potential sites for the qualitative phase, but four sites were purposefully chosen based on their high scores on variables related to self-disclosure during the quantitative phase. As the study started with a survey and was followed by interviews and focus group discussions, the interview and focus group discussion participants were selected from the population of participants who had responded to the survey. Focus group discussions are a technique whereby participants are brought together to discuss a given issue with the purpose of gathering insight from the participants’ complex personal experiences, beliefs, perceptions, and attitudes through a regulated interaction (Nyumba, Wilson, Derrick, & Mukherjee, 2018). Participant characteristics that were considered when selecting the ALHIV for FGDs were gender, age, ethnic background and social background. However, the FGDs were not entirely homogenous because they were made up of both genders, which promotes honest and spontaneous views. The sample size for all participants was not pre-determined, but data were collected until saturation was reached, and there was no new data that emerged to add any further insights, but there was repetition of themes (Vasileiou et al. 2018). Table 2 shows the sample size in the qualitative phase.

**TABLE 2:** Sample size for adolescents, nurses and policymakers in the qualitative Phase (*N* = 51).

Identity	Sample size		
	Adolescents (*n* = 23)	Nurses (*n* = 24)	Policymakers (*n*= 4)
Site A	5	6	-
Site B	7	6	-
Site C	5	7	-
Site D	6	5	-
Policymaker A	-	-	1
Policymaker B	-	-	1
Policymaker C	-	-	1
Policymaker D	-	-	1

*Source*: Adapted from Dlamini, B.P. & Mtshali, N.G., 2024a, ‘Nurses and policymakers role in preparing adolescents with HIV for self-disclosure in Eswatini’, *African Journal of Primary Health Care Family Medicine* 16(1), 4332. https://doi.org/10.4102/phcfm.v16i1.4332

### Data collection

Because of the nature of the study, data were collected in two phases; initially, quantitative data were collected, followed by qualitative data. A 99-item self-administered questionnaire divided into 10 sections was used to gather quantitative data between April and September 2022. The sections included: demographics (14 items), experiences of adolescents disclosing their HIV status (16 items), relationships with family and non-family members (4 items), learning about tools to support HIV status disclosure (19 items), self-disclosure of HIV status (8 items), safer sex self-efficacy (5 items), disclosure self-efficacy (5 items), HIV-related stigma (9 items), sexual and reproductive health (9 items) and adherence to ART (10 items). Standardised instructions when filling out the self-administered questionnaire were adhered to thus maintaining uniformity in the collection of data. Questions that appear on the questionnaire were adapted from previous studies. HIV status disclosure and disclosure efficacy questions were adapted from a questionnaire by Kidman and Violari (2020). Questions on learning about tools to support HIV disclosure were adapted from Okawa et al. (2017). Questions on safer sex self-efficacy were adapted from Nöstlinger et al. (2015). Questions on sexual and reproductive health were adapted from Deribe et al. (2008).

Age, gender, biological parents’ status, alive or deceased, and whether or not they were on ART were recorded demographic variables associated with HIV together with the age at which the participant was first informed of their HIV status and the individual who disclosed it, along with the location of the disclosure. Participants were asked if they had ever told a close friend, boyfriend or girlfriend they were living with HIV. Those adolescents who had not disclosed were asked to list the reasons they felt uncomfortable disclosing, with possible answers being ‘not yet the right time’, ‘not knowing how to disclose’ or ‘fear of rejection’. After disclosing, adolescents were asked to choose from three possible responses from friends, boyfriends or girlfriends: rejective, not rejective but not supportive or supportive.

Participants were also asked if they were provided with information to promote disclosure, and they answered questions like, ‘Have you ever been educated on how to tell someone that you are living with HIV?’ with a yes or no. Share confidential information with a partner in a romantic relationship? How to prevent spreading HIV to a sexual partner? A modified version of the scale (Kalichman et al. 2001), which ranges from 1 (strongly agree) to 4 (strongly disagree), was used to determine disclosure self-efficacy and statements like ‘I am certain that I could tell a new partner about my HIV status before having sex even if I don’t know that person’s HIV status’ plus ‘I feel confident telling someone I was dating that I am HIV positive’. Enquiries were made to the participants regarding their sexual history, including whether or not they had disclosed their HIV status prior to engaging in sexual activity, whether they had ever used a condom during that first encounter and whether or not they had performed so consistently over the previous 12 months. They were asked to respond with a yes, no or don’t remember the response to each of these questions. The questions on sexual behaviour assisted the researchers in comprehending the intricate interplay of factors that influence sexual behaviour and its outcomes. Furthermore, data from these questions were analysed to identify patterns, risk factors and relevant solutions and ultimately model development. Qualitative data in ALHIV were collected through individual semi-structured interviews, which lasted about 30 minutes, utilising interview guides in both languages, English and siSwati. Prior to the collection of data, permission was granted by participants to be recorded using a portable audio recorder. To facilitate and encourage opening up of the participants, the individual interviews were conducted in a designated room in the health facilities away from anticipated disturbances and at a time most comfortable and convenient for the participants. A social worker was on standby during the interviews of adolescent participants in all the chosen sites for a debriefing session if they needed it. A total of three FGDs were held, and each FGD was made up of 5–6 participants of mixed gender to enhance the quality of discussions and their outcomes brought about by different ideas and perspectives. The length of each FGD was approximately 45 min, and participants were seated in a semi-circle to encourage face-to-face interaction. A semi-structured interview guide with open-ended questions was used to guide the discussion, to enable probing and to elicit more data from the participants. Literature from previous studies with similar topics guided the development of the interview guides. The tools were piloted with two participants from each group who were not part of the main study to determine their efficacy, and this resulted in insignificant changes to the tools.

For nurses, interviews were conducted in both English and siSwati, and questions asked were about their experiences with HIV self-disclosure to ALHIV, have they been trained on self-disclosure counselling and the steps they followed in preparing adolescents for disclosure. Policymakers’ interviews were conducted in English, and they lasted approximately 45 min. Questions posed were the availability of sufficient funds for HIV prevention programmes, the availability of national self-disclosure guidelines for ALHIV and the training of nurses in providing comprehensive adolescent services.

### Data analysis

The Statistical Package for Social Sciences (SPSS) version 28 was used to analyse quantitative data. The participants’ socio-demographics were summed up using descriptive statistics, and factors related to HIV status disclosure were identified through bivariate analysis. The most significant independent variable associated with disclosure was identified through logistic regression analyses using odds ratios (OR) and 95% CI. To analyse qualitative data, grounded theory was used, and in grounded theory, data collection, analysis and the subsequent emerging model are intertwined (Corbin & Strauss 2015). Conducting a grounded theory research study is not linear, but rather it is iterative and recursive (Chun Tie, Birks & Francis 2019). Therefore, there was concurrent data collection and data analysis, through the various stages of coding, constant comparative analysis and theoretical sampling, which led to adjustment of the interview guide as required (Chun Tie et al. 2019). The recordings were transcribed verbatim and then translated into English. Initially, open coding was performed, whereby transcripts were read until the researcher got a sense of the data. The transcripts were read line by line, and key phrases were identified and highlighted to uncover the participants’ thoughts, ideas and meanings (Corbin & Strauss 2015). The data were compared to look for similarities and differences in patterns, and many codes were inductively generated (Charmaz 2006).

Thereafter, concepts that were similar or related in meaning were grouped into subcategories and categories in terms of their properties and dimensions. Once categories began to develop, the researchers moved on to the next stage of axial coding. At this point, the researchers then integrated quantitative and qualitative findings. Emergent categories were then closely examined and compared for similarities and differences (Corbin & Strauss 2015). In axial coding, categories are related to their subcategories to form more precise and complete explanations of the phenomena under study (Corbin & Strauss 2015). During the analytic stage of axial coding, the researchers engaged in constant comparative analysis, leading to the refinement of categories (Chun Tie et al. 2019) as presented in Table 3. Selective coding, the final step followed, where the central category HIV self-disclosure was identified and became the focus of analysis. Thereafter, the analytic power of the central category was further examined to determine how it related to the other categories to form an explanatory whole (Corbin & Strauss 2015).

**TABLE 3:** Concept reduction and refinement.

Concepts	Sub-concepts
**Adolescent empowerment**	
Adaptation	Internalised HIV status Strong self-identity Self-regulation
Resources	Educational materials Disclosure guide
Precursors	Disclosure readiness Self-disclosure efficacy
Disclosure event	Dynamic, ongoing process Verbal or non-verbal Content Reaction
Follow-up	Post-disclosure impact Education
Feedback loop	Monitoring
**Enablers**	Self-disclosure guidelines
	Capacity building
**National HIV strategic framework**	HIV self-disclosure policy
	Funding for HIV self-disclosure policy implementation
	Multi-sectoral commitment

*Source:* Adapted from Dlamini, B.P. & Mtshali, N.G., 2024a, ‘Nurses and policymakers role in preparing adolescents with HIV for self-disclosure in Eswatini’, *African Journal of Primary Health Care Family Medicine* 16(1), 4332. https://doi.org/10.4102/phcfm.v16i1.4332; Dlamini, B.P. & Mtshali, N.G., 2024b, ‘Views of adolescents living with perinatally acquired HIV on HIV status disclosure in Eswatini’, *AIDS Care* 36(9), 1263–1271. https://doi.org/10.1080/09540121.2023.2299334

The storyline, as the conceptualisation of the core category, was developed, and it allowed in-depth descriptions of the categories and ensured that the final theory was grounded in the data. Furthermore, it provided a theoretical framework to enhance understanding, thus explicating the theory (Chun Tie et al. 2019). Conceptualisation further contextualised the research by providing a narrative framework that demonstrated how the different components of the study interconnect and can be presented in a coherent manner. After conducting a focused literature search, diagrams were used to integrate concepts and the relationships among them although these were not written in a cause-and-effect manner, but they focused on those concepts that reached the status of major categories (Corbin & Strauss 2015). The advantage of a focused literature review was that it examined in depth a particular field of adolescent HIV self-disclosure and went deeper into its application and implications because of its more limited scope. Finally, the model was presented in the form of a diagram to show how concepts interrelate.

### Study rigour

The items used in the questionnaire in this study were adapted from previous questionnaires that have been tested and have been proven reliable in previous similar research (Babbie 2016) with Cronbach’s alpha (α) value ranging above 0.7, thus demonstrating acceptable reliability (Creswell & Creswell 2018). For the qualitative phase, using Lincoln and Guba’s model, four trustworthiness criteria: credibility, dependability, confirmability and transferability (Lincoln & Guba 1985), improved the study’s credibility. Extended periods of interaction between the participants and researchers before data collection began allowed participants to become acquainted with them. This was performed by engaging with participants during recruitment and thereafter on two other occasions in informal conversations before conducting formal interviews to establish rapport and gain their trust. The use of a semi-structured interview guide also allowed flexibility during the interviews, thus allowing the researchers to probe deeper when necessary (Forero et al. 2018). Dependability was ensured by reporting all activities that occurred during the study. An audit trail was established, whereby a detailed track record of the process of data collection was developed, and the transcripts were re-read against the audio files for accuracy during data analysis. Confirmability was ensured by tape-recording data collection, writing field notes and writing a research report. A reflective journal was also kept throughout the data collection process, and it assisted in capturing relevant and useful information. Transferability was ensured by utilising purposive sampling techniques to make sure that the selected participants were a true representation of the population under study and also by providing a detailed description of the number of participants who met the inclusion criteria and were part of the study.

### Data management and storage plan

Currently, completed questionnaires are secured at the researcher’s office in a lockable cupboard. With the participants’ consent, all of the interviews and focus group discussions were recorded using a portable audio recorder. In order to ensure accuracy during transcription and to avoid omitting any important data, field notes were also taken throughout the interviews. All transcribed interviews are stored in a laptop secured with a password. During transcription, no personal identifiers appeared on the documents, but all participants were allocated pseudonyms.

### Ethical considerations

This study was carried out with approval from the country’s Health & Human Research Review Board (EHHRRB064/2021), the Higher Degrees Ethics Committee at a Public University (BREC/00002527/2021) and the sites where data collection took place. Voluntary participation was upheld in this study, which meant participants were free to withdraw at any time. Confidentiality, privacy and anonymity were respected during the data collection process. Privacy was maintained in the process of selecting ALHIV by approaching them to participate in the study through ‘Teen Clubs’, which is a special clinic only for ALHIV. All participants signed consent forms before data collection, and adolescents below the age of 18 years signed assent forms while their parents signed consent forms.

## Results and discussion

This article focuses on the development of the model as other findings have already been reported in preceding articles. Previous findings in our study revealed a low rate of HIV status self-disclosure (22%) by Swati ALHIV, and those adolescents who had high HIV knowledge were two times more likely to disclose their status than adolescents who had low HIV knowledge. Furthermore, it was challenging for ALHIV to accept their status, as they were still grappling with living with HIV, and this made onward disclosure difficult. A majority of nurses and policymakers clarified that to self-disclose, ALHIV require ongoing support, both immediately following the disclosure and thereafter, to help them manage living with HIV (Dlamini & Mtshali 2023, 2024a, 2024b).

The six elements of Chinn and Kramer (2011) for theory development were used to develop this model. These elements comprised: (1) the purpose for which the model is developed and stipulates the context as well as the situation of application; (2) identification and classification of model concepts, which are defined as groups of words and symbolic representations of reality that serve as building blocks of a theory; (3) assumptions of the model, which are the basic acknowledged truths that are considered as essential to theoretic or conceptual reasoning; (4) operational definition of concepts to explain the meaning of the concepts in the context of the model being developed; (5) nature of relationships, which describe how concepts are linked together to provide the whole structure and (6) stating the structure of the model, which gives the complete form to the conceptual relationships within it.

### Purpose of the model

The primary purpose of the model was to: (1) offer a framework to be utilised as an instrument in assisting ALHIV to self-disclose their status, (2) provide nurses with a guide on how to facilitate self-disclosure in ALHIV and (3) add to the body of knowledge in ALHIV self-disclosure.

### Assumptions of the model

The assumptions for this adolescent self-disclosure model have originated from the results of the study. They are: (1) adolescents need to accept their HIV status and learn to live with HIV, (2) adolescents need to be emotionally ready before they can disclose, (3) comprehensive HIV education is fundamental in preparing ALHIV for disclosure, (4) disclosure is a dynamic ongoing process (5) nurses’ support is essential during the disclosure process and (6) post-disclosure communication is necessary to ascertain effects of disclosure and educate about HIV.

### Concept identification and definition

Concepts are regarded as the most critical components that are considered when generating theory based on the premise that they guide enquiry (Walker & Avant 2011). The central concept for this model is HIV (status) self-disclosure. There are four key concepts related to the central concept, and these are: (1) national HIV strategic framework, (2) enablers, (3) adolescent empowerment and (4) outcomes. All the key concepts have sub-concepts under them, which will be discussed later in the article. The central concept of HIV self-disclosure is defined as voluntary sharing of the status by an ALHIV with another person for the purpose of this model. Disclosure can afford individuals a chance to share thoughts and feelings and enhance intimacy within personal relationships (Chaudoir & Fisher 2010). Furthermore, self-disclosure has various public health benefits, especially for sexual partners, as it increases the chances of condom use and safer sex practices, as well as stops the spread of HIV (Thoth et al. 2014).

*The National Strategic Framework (NSF)* refers to a tool that provides strategic direction for the national response towards HIV disclosure using specific and integrated plans. The NSF should have a clearly articulated vision, goals and objectives and further identify key priorities and strategies to achieve the set targets. Streamlining actions towards successful self-disclosure will make the national response more effective. This key concept includes three sub-concepts: (1) HIV self-disclosure policy, (2) funding for HIV self-disclosure policy implementation and (3) multi-sectoral commitment.

HIV self-disclosure policy refers to a legal document that directs the action of national key players involved in endorsing HIV self-disclosure. Through the commitment of the MoH, the right to autonomous HIV disclosure should be upheld during service delivery to ensure the services remain relevant and responsive to the needs of ALHIV. Pertinent issues that should be addressed by the self-disclosure policy are: age of self-disclosure of the adolescent, nurses’ self-disclosure training, availability of services at all government sites providing HIV care and management, connecting ALHIV to peer networks, availability of self-disclosure guidelines, debriefing sessions, pre-disclosure and post-disclosure support, patient monitoring and periodic quality control checks (Dasgupta et al. 2016).

Funding for HIV self-disclosure policy implementation requires mobilising the resources required for full implementation, and this necessitates the MoH to collaborate with the government in mobilising additional funds and technical expertise to implement the self-disclosure policy. The government funding can be augmented through reduced budget allocation to have surplus funds and supportive information exchange in coordinating structures and devising a strategy to have a focal entry point for supporting HIV self-disclosure interventions in the country to avoid duplication and thus ensure sustainability.

Multi-sectoral commitment in this study is regarded as a collaborative approach spanning across government, non-governmental organisations and community groups with a mutual goal of addressing HIV self-disclosure. When properly co-ordinated, multisectoral collaboration drives the process, unites members, facilitates and directs the development of the implementation plan with activities and time frames that are aligned to the NSF while monitoring and appraising progress on how the plan is effected (Mahlangu et al. 2017). Multisectoral commitment is important because, increasingly, it is recognised that no single sector can address the multiple drivers and impacts of HIV and AIDS (Kar 2014) and that integrated, multi-level efforts by government, working together with other sectors, are necessary, which has led to many countries in sub-Saharan Africa having adopted the multi-sectoral approach in their national HIV and AIDS plans (Mahlangu et al. 2017).

*Enablers* are factors and conditions whose availability supports the implementation of HIV self-disclosure. In this study, enablers are: (1) self-disclosure guidelines and (2) capacitation of nurses and community health workers (CHWs). HIV self-disclosure guidelines refer to statements that comprise recommendations developed using a systematic process based on prevalent guideline development standards. They should be developed by the MoH in collaboration with relevant stakeholders (government, private entities, nurses and CHWs) and then cascaded down to facilities for implementation. For the HIV self-disclosure guidelines to be effective, there should be comprehensive consultation during the development phase to ensure success and uptake. Pre-disclosure assessment, self-disclosure algorithm and post-disclosure support and linkage should be included in the self-disclosure guidelines.

Capacity building refers to equipping nurses and CHWs with the necessary skills to facilitate self-disclosure. In this study, this can be achieved through the scale-up of in-service training and sensitisation of nurses resulting in qualified personnel who have been trained on adolescent HIV self-disclosure and have demonstrated characteristics such as being committed to providing adolescent-friendly services, act as a mentor, supporter, facilitator, critical thinker and monitor throughout the disclosure process. Because of the government being financially constrained, more sustainable interventions with domestic funding by the government can be implemented to collect taxes efficiently and reduce expenditure to have surplus funds as this would facilitate progress toward financial autonomy and reinforcement of ownership of public policy and the ability to mobilise funds to train the nurses to ensure they have the required skills. Nurses’ training is integral because nurses are the primary health workers responsible for managing HIV care in low-resourced countries like Eswatini (Knettel et al. 2021).

Community health workers in this study are referred to as non-professional health workers who have been trained in adolescent HIV and AIDS management and are currently facilitating the ‘teen clubs’ for ALHIV in facilities. While they have no formal professional certificate in tertiary education (Mwai et al. 2013), the availability of CHWs reduces the burden of care placed on the nurses (Knettel et al. 2021). In the context of this study, the supportive role of CHWs entails maintaining regular contact with the adolescents to provide ongoing ART adherence education and disclosure counselling, scheduling monthly follow-up appointments and assisting in tracking ALHIV who may be lost to care. The CHWs also facilitate peer support by asking those adolescents who have gone through successful disclosure to coach or mentor other adolescents and support them in their disclosure. Adolescents are a unique population, and the CHWs need to utilise open communication, have a collaborative relationship with the ALHIV to realise mutually established goals and be flexible when providing care to be able to engage them productively and promote retention in care.

*Adolescent empowerment* refers to the process of equipping ALHIV for self-disclosure. This major concept is supported by six sub-concepts, which are: (1) adaptation, (2) resources, (3) precursors, (4) disclosure event, (5) follow-up and (6) feedback loop.

The sub-concept adaptation refers to the achievement of a significant change in the life of the ALHIV since their HIV diagnosis. This change should enhance well-being in the life of the ALHIV and will be characterised by an internalised HIV status, which refers to the acceptance of living with a chronic manageable condition, a strong self-identity that entails being self-aware and valuing a unique identity despite living with HIV and having goals and values to assist in disclosure decision making. Self-regulation is defined as a person’s capacity to control their thoughts, behaviour and reasoning processes (Billore, Anisimova & Vrontis 2023) and is regarded as an important part of overall mental and physical well-being. In the context of self-disclosure, emotional self-regulation is crucial because it will enable ALHIV to react appropriately in stressful situations.

The sub-concept resources denote educational materials such as tools to be used for the purpose of preparing the adolescent for self-disclosure. Before the adolescent can disclose, they need to have extensive HIV knowledge, and the nurse should encourage the adolescent to learn relevant information about HIV and how to live positively with HIV so that they can feel empowered with the right knowledge and be able to share it with others (Khan et al. 2023). The preparative tools should contain HIV information that includes what HIV is, how it is transmitted and the role of ART in keeping an individual living with HIV healthy. Additionally, ALHIV should be educated about Pre-Exposure Prophylaxis (PrEP) which is daily oral medication used to prevent HIV infection (Golub 2018), the concept of Undetectable = Untrasmittable (U = U), HIV transmission hazards and the role condoms play in the prevention of Sexually Transmitted Infections (STI’s) and HIV. Sexually Transmitted Infections (STI’s) are caused by a wide variety of bacteria, viruses, and parasites that spread from one person to another through vaginal, anal, or oral sexual contact (Wagenlehner et al. 2016). To help the adolescent feel prepared, drafting a disclosure guide that outlines what will be said and providing role-playing opportunities for preparing potential questions and responses should be performed (Elizabeth Glaser Pediatric AIDS Foundation 2018). Providing adolescents with pertinent information when assisting them in disclosing their HIV status is crucial, as it would enable them to respond appropriately to inquiries from those they are sharing their status with.

Precursors in this study are referred to as situations or phenomena that precede successful self-disclosure. Disclosure readiness implies that ALHIV should only disclose when they are comfortable with sharing their status. To determine the state of readiness, a disclosure readiness assessment should be conducted, whereby the potential benefits and disadvantages of disclosure are reviewed, and ALHIV are reminded that HIV status disclosure is an individual choice on when, how and whom to reveal their status to and their state of preparedness to deal with either positive or negative outcomes (Elizabeth Glaser Pediatric AIDS Foundation 2018). Self-disclosure efficacy refers to having the ability to disclose, which comes about as a result of the ALHIV being given all the resources and skills that will facilitate disclosure, as it may be daunting, especially for the first time.

Disclosure event refers to the encounter when the HIV status is being shared with the chosen confidant. In the context of this model, disclosure is a dynamic, ongoing process rather than a single event. Disclosure happens for various reasons, and these are to protect the partner, strengthen the relationship or because the partner feels they have a moral obligation to disclose (Chaudoir & Fisher 2010). The ALHIV can choose to use verbal or non-verbal communication to disclose the status, and when using verbal communication, the disclosure will be face to face, whereas in non-verbal communication, short text messages and WhatsApp messages can be used (Dlamini & Mtshali 2023; Mlilo et al. 2020). Content refers to the amount of information that will be shared about the HIV status. The ALHIV can share basic facts initially and then gradually add more information regarding their HIV status. Regardless of the method chosen for disclosure, it is imperative to utilise concise explanations of the illness. If face-to-face disclosure is used, HIV-related materials should be carried along to address any questions the person receiving the disclosure may have and to clarify the facts. When the person to whom disclosure has been made acknowledges that they comprehend the conveyed information that the ALHIV is living with HIV, then disclosure will be considered as having taken place (Chaudoir & Fisher 2010). Reaction refers to the observed behaviour of the confidant towards the ALHIV after disclosure, which can either be negative or positive. Negative reactions include fights, blame and rejection and positive reactions are hugging, understanding and acceptance (Damian et al. 2019; Galano et al. 2017). Negative reactions may deter future disclosures, whereas positive reactions may encourage disclosure to more people.

Follow-up refers to the post-disclosure period, whereby the adolescent will initiate communication with the confidant. The objective of post-disclosure follow-up is to identify the perception and the degree of coping of the confidant with the HIV status and further educate about HIV based on questions that will arise. The ALHIV should ascertain how the disclosure may have altered the dynamics of the relationship and how this might be negotiated until normal relations are restored, as an assessment of the post-disclosure impact (Elizabeth Glaser Pediatric AIDS Foundation 2018). During this time, the adolescent should engage in open discussion and additional education to dispel common misconceptions and presumptions about HIV (Hogwood et al. 2013), and the confidant should be encouraged to find out more information on their own, with considerations that they may need space and time to reflect on the disclosure.

The feedback loop refers to a mechanism through which ALHIV provide information to the nurses on the disclosure process. Nurses should monitor the disclosure process through the feedback loop, find out how it transpired and provide post-disclosure support on how to handle the psychological effects of self-disclosure (Khan et al. 2023). An appointment for the adolescent to return to the facility and provide feedback while their feelings and thoughts are still new should be scheduled prior to disclosure. The nurse should determine the overall experience of disclosure, ask about any difficulties they encountered and then find out the reactions of the person they disclosed to and how this resonated with them. To ensure continuity of treatment, the nurse should also record the disclosure event in the adolescent’s file and monitor the adolescent’s well-being during the post-disclosure period (Elizabeth Glaser Pediatric AIDS Foundation 2018).

### Model outcomes

In this study, HIV self-disclosure outcomes were made up of the following sub-concepts: (1) individual outcomes and (2) healthcare system outcomes.

### Individual outcomes

Sub-concepts that emerged as individual-related outcomes were: (1) improved quality of life and (2) enhanced psychosocial support.

Improved quality of life in this model refers to the enhanced physical and mental well-being of ALHIV as a result of disclosure. In this study, the enhanced physical well-being will be a result of adherence to ART and retention in care as these bring about good clinical outcomes. Conversely, enhanced mental well-being will be a result of ALHIV having decreased anxiety because there will be no need to hide the HIV status, thus reducing stress and lowering risky sexual behaviours (Irfantoro & Rukmi 2020).

Enhanced psychosocial support will be the positive result of status disclosure for ALHIV, and in this study, social support symbolises a structure where vulnerable adolescents get assistance from significant others and their immediate social networks (Lan et al. 2015). The belief is that adolescents who receive social support post-disclosure will have improved self-esteem and better coping mechanisms and attain a healthier lifestyle (Biraguma, Mutimura & Frantz 2018).

### Healthcare system outcomes

The following sub-concepts emerged as health system-related outcomes: (1) quality care services, (2) increased patient advocacy and (3) reduced new adolescent HIV infections.

Quality care services refer to healthcare services that cater to the specific needs of the ALHIV. Nurses will be competent to provide quality care as a result of the knowledge and skills acquired after undergoing in-service training and taking part in the development of HIV self-disclosure guidelines for ALHIV. Furthermore, nurses need to be empowered on how to provide adolescent-friendly services because if they are not empowered, they will render substandard care, leading to adolescent dissatisfaction and poor health outcomes; however, empowerment through training strengthens the current health structures to properly accommodate ALHIV.

Increased patient advocacy in this study refers to nurses’ safeguarding of ALHIV vulnerability as their clients who do not have full control over their care. Nurses need to demonstrate patient advocacy by allowing ALHIV to make decisions without coercion, respect their individuality and humanity, maintain patient privacy and always act in the adolescents’ values and best interests (Abbasinia, Ahmadi & Kazemnejad 2020). Furthermore, nurses must increase their participation in healthcare policy-making to improve healthcare policies, facilitate access to health care and enhance the overall well-being of ALHIV and society as a whole (Abbasinia et al. 2020).

Reduced new adolescent HIV infections will be outcomes that are realised after nurses and CHWs undergo in-service training to empower them with knowledge on how to facilitate self-disclosure and also through the implementation of self-disclosure policies and guidelines. Ultimately, this will lead to increased self-disclosure by ALHIV to others and especially sexual partners, thus reducing onward transmission of HIV.

### Nature of relationships between concepts and structural model description

Concept development is a key component when developing a model (Walker & Avant 2011). The concepts in this model were developed from the study results and the concepts and relationships between concepts laid the foundation for developing the model. In this model, ‘HIV self-disclosure’ is the central concept, and it has key concepts that are directly and indirectly related to it, and these are: (1) national HIV strategic framework, (2) enablers, (3) adolescent empowerment and (4) outcomes. The key concept *national HIV strategic framework* is directly linked to the central concept of HIV self-disclosure, and it lays the foundation for ALHIV self-disclosure through the enactment of an *HIV self-disclosure policy* by mobilising *funding for HIV self-disclosure policy implementation* from domestic funding and lobbying for *multi-sectoral commitment* by inviting relevant stakeholders to join forces in promoting adolescent self-disclosure. The key concept *enablers* are directly linked to the NSF and focus on building capacity for personnel who will efficiently prepare ALHIV to disclose and the establishment of self-disclosure guidelines, which will assist nurses and CHWs during implementation.

**FIGURE 1:**
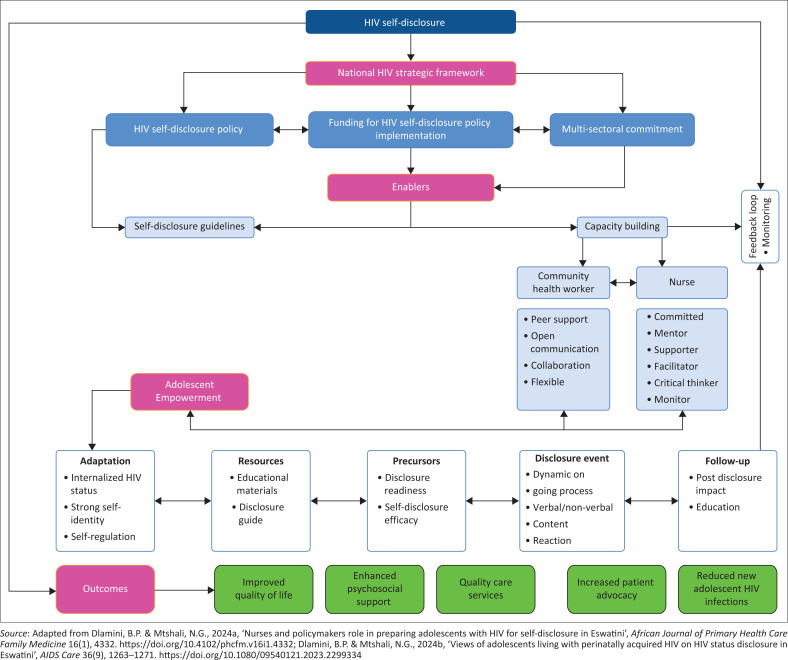
Adolescents living with HIV self-disclosure model.

The key concept of adolescent empowerment is indirectly linked to enablers and is made up of various sub-concepts that characterise the different steps in the process of self-disclosure. *Adaptation* is the initial step, which requires the ALHIV to normalise living with HIV without feeling different from their peers. *Resources* are then used to assist in preparing adolescents for disclosure, and these give practical guidance on how the adolescent can actually go through the process. *Precursors* then prompt the adolescent to disclose because they are ready to disclose and the *disclosure event* is the actual disclosure encounter where the status is revealed. This disclosure event is flexible because the adolescent is not required to reveal all information at once, but as an ongoing dynamic process, it should be allowed to unfold in a way that suits the adolescent and chosen confidant. Post-disclosure *follow-up* is performed by the adolescent to determine how the disclosure was received, and it also gives a chance to further educate about HIV. The *feedback loop* requires the adolescent to report back to the nurse about their disclosure experience and further affords the adolescent a chance to get post-disclosure support. The arrows between all the sub-concepts are two sided, meaning that the process is not linear, but adolescents can move freely between steps until there is absolute certainty that they are ready for the next step. Figure 1 displays the self-disclosure model for adolescents with perinatally acquired HIV in Eswatini.

### Recommendations

The researchers recommend that the active use of this model by nurses is crucial to facilitate and improve the self-disclosure rate among ALHIV. This model should be communicated to adolescents with perinatally acquired HIV to equip them with the necessary skills to enable HIV status disclosure. Furthermore, this model can be utilised by adolescents with behaviourally acquired HIV. The researchers also advocate for optimal emotional support for adolescents’ post-disclosure as they deal with the consequences of disclosure until they regain a sense of normalcy again.

### Strengths and limitations

The strength and major contribution of this study is that it is the first of its kind to develop an adolescent HIV self-disclosure model in Eswatini. This study also employed a mixed methods design to provide a more complete understanding of the research problem under investigation. The limitation of the study is that it was conducted in Eswatini context and findings cannot therefore be generalised to other contexts.

## Conclusion

Adolescents living with HIV have challenges in disclosing their HIV status to others because of various reasons. The presented model has explicitly shown that self-disclosure cannot occur until there is a readiness to disclose. Moreover, disclosure requires the ALHIV to be well-informed about HIV before they can be able to efficiently execute this challenging process. In this model, nurses’ supportive role is essential, and they should, as primary healthcare givers, discuss with ALHIV the advantages and benefits of self-disclosure.
